# Dr. J. Stone Armstrong

**Published:** 1901-04

**Authors:** 


Dr. J. Stone Armstrong, of Buffalo, died at his home March 3, 1901, aged 61 years. He graduated at Jefferson Medical College in 1879, and had been a resident of Buffalo for about twenty years. His body was incinerated at his request in the Delavan Avenue Crematory.

